# Influenza and COVID-19 Vaccination Coverage Among Health Care Personnel — United States, 2024–25 Respiratory Virus Season

**DOI:** 10.15585/mmwr.mm7512a2

**Published:** 2026-04-02

**Authors:** Mehreen Meghani, Zhuping Garacci, Hilda Razzaghi, Marie A. de Perio, A. Scott Laney, Jennifer L. Kriss, Carla L. Black

**Affiliations:** ^1^Immunization Services Division, National Center for Immunization and Respiratory Diseases, CDC; ^2^Cherokee Nation Operational Solutions, Tulsa, Oklahoma; ^3^Office of the Director, National Institute for Occupational Safety and Health, CDC; ^4^Division of Respiratory Health, National Institute for Occupational Safety and Health, CDC.

**Summary**

**What is already known about this topic?**

Influenza and COVID-19 cause considerable morbidity, including among health care personnel (HCP). For the 2024–25 respiratory virus season, the Advisory Committee on Immunization Practices recommended influenza and COVID-19 vaccination for all HCP.

**What is added by this report?**

During the 2024–25 respiratory virus season, HCP influenza and COVID-19 vaccination coverage rates were 76.3% and 40.2%, respectively. Among HCP whose employer offered on-site influenza and COVID-19 vaccination, coverage was higher (73.0% and 42.9%, respectively) than it was among those whose employer did not offer on-site vaccination (41.4% and 19.8%, respectively).

**What are the implications for public health practice?**

Increasing vaccination coverage by implementing workplace policies including offering on-site vaccination might increase coverage and reduce influenza- and COVID-19–related morbidity among HCP.

## Abstract

The Advisory Committee on Immunization Practices (ACIP) recommends that all health care personnel (HCP) receive an annual influenza vaccination to reduce the risk for influenza and influenza-related morbidity and mortality among themselves and their patients. For the 2024–25 respiratory virus season, ACIP also recommended that all HCP be vaccinated against COVID-19. During March 26–April 17, 2025, CDC conducted a nonprobability opt-in internet panel survey of 2,650 U.S. HCP to estimate influenza and COVID-19 vaccination coverage during the 2024–25 respiratory virus season. Overall, 76.3% of HCP reported having received an influenza vaccine and 40.2% reported receiving the 2024–25 COVID-19 vaccine. Influenza vaccination coverage was highest among pharmacists (94.6%), physicians (92.6%), and HCP who worked in hospital settings (88.3%). COVID-19 vaccination coverage was highest among physicians (46.7%), assistants or aides (46.7%), and HCP who worked in long-term care and home health care settings (44.5%). Both influenza and COVID-19 vaccination coverage rates were highest among HCP whose employer required or recommended the vaccines or offered them on-site. A multipronged approach, including educating HCP about benefits of vaccination and implementing workplace strategies (such as employer vaccination recommendations or offering on-site vaccination) might improve vaccination coverage and reduce influenza- and COVID-19–related morbidity among HCP.

## Introduction

Health care personnel (HCP) are at risk for exposure to respiratory infections, including influenza and COVID-19 (*1*,*2*). Receipt of influenza and COVID-19 vaccination by HCP helps reduce morbidity among HCP as well as workplace absenteeism (*3*,*4*). In addition, preventing influenza among HCP through vaccination might help prevent influenza among patients in health care settings by reducing their exposure (*4*). The Advisory Committee on Immunization Practices (ACIP) recommends annual influenza vaccination for all HCP (*5*). During the 2024–25 respiratory virus season, ACIP also recommended that all HCP be vaccinated against COVID-19 (*6*). Data from a nonprobability opt-in internet panel survey of 2,650 U.S. HCP were analyzed to evaluate workplace vaccination requirements and estimate influenza and COVID-19 vaccination coverage during the 2024–25 respiratory virus season.

## Methods

### Data Source

An internet panel survey of U.S. HCP was conducted during March 26–April 17, 2025, to provide estimates of influenza and COVID-19 vaccination coverage among HCP during the 2024–25 respiratory virus season. Similar annual surveys have been conducted since the 2010–11 influenza season (FluVaxView | CDC) (*7*). For each annual survey, respondents were recruited from two preexisting national opt-in internet sources: Medscape,* a medical website managed by WebMD Health Professional Network, and general population internet panels operated by Dynata.^†^ Panel respondents were eligible to participate based on screening questions related to health care work setting and occupation. Respondents could select one or more work settings, and, based on their selections, were categorized into four work settings: 1) hospital, 2) ambulatory care, 3) long-term care (LTC) (including nursing homes, assisted living facilities, other long-term care facilities, home health agencies, and home health care settings), and 4) other (dentist’s offices or dental clinics, pharmacies, emergency medical services, and other settings where clinical care or related services are provided to patients).

### Data Analysis

Responses were weighted to the distribution of the U.S. population of HCP^§^ by occupation,^¶^ age, sex, race and ethnicity, work setting, and U.S. Census Bureau region. A poststratification weight for each respondent in the survey was calculated by fitting a generalized exponential model and estimating the model parameters using calibration equations, which calibrate the sample to the target population and minimize the unequal weighting effect.

Weighted influenza and COVID-19** vaccination coverage for the 2024–25 and 2023–24 seasons (and corresponding 95% CIs) were estimated for each work setting, occupation, and demographic characteristic. Each participant was asked four separate questions to ascertain employer vaccination recommendations or requirements and on-site influenza and COVID-19 vaccination.^††^

To examine trends in HCP influenza coverage over time, annual influenza vaccination coverage for all work settings from the previous 10 seasons from similar annual surveys conducted since the 2015–16 influenza season were examined. The Korn-Graubard method was used to calculate CIs around coverage estimates, assuming that the weighted estimates were unbiased. CDC’s reliability criteria for proportions were applied to the coverage estimates in the descriptive analyses of HCP characteristics (*8*). T-tests were used to determine differences in estimated influenza and COVID-19 vaccination coverage among subgroups. Rao-Scott chi-square testing was used to calculate differences in estimated influenza vaccination coverage between seasons; p-values <0.05 were considered statistically significant. SAS/STAT survey procedures (version 9.4; SAS Institute) were used to conduct all analyses. This activity was reviewed by CDC, deemed not research, and was conducted consistent with applicable federal law and CDC policy.^§§^

## Results

Among 2,807 initial participants in the 2024–2025 survey, 2,702 (96.3%) completed the survey^¶¶^; among these, 52 participants were excluded because they reported working in a setting other than those listed, and the description did not qualify as a health care setting. The final analytic sample included 2,650 respondents.

### Influenza Vaccination Coverage

**FIGURE 1:**
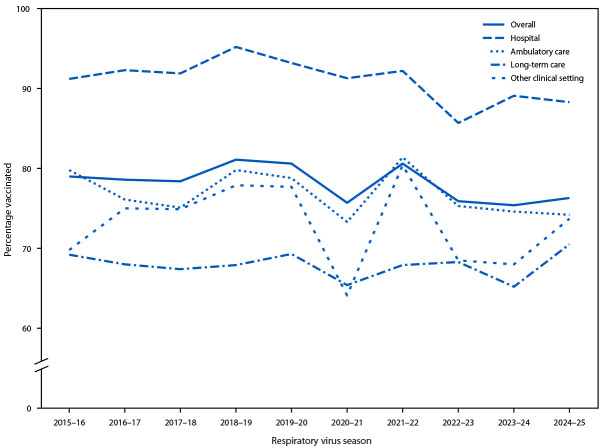
Percentage of health care personnel who received an annual influenza vaccination,* by work setting^†^ — internet panel surveys,^§^ United States, 2015–16 through 2024–25 respiratory virus seasons * Before the 2020–21 influenza season, weights were calculated based on the known population control totals of the U.S. population of health care personnel, controlling for the main effects (occupation, age group, race and ethnicity, sex, work setting, and U.S. Census Bureau region); for 2020–21 and later seasons, interaction terms between occupation and the other main effects were added in the weighting model (Influenza Vaccination Coverage Among Health Care Personnel — United States, 2020–21 Influenza Season | FluVaxView | Seasonal Influenza (Flu) | CDC). ^†^ Respondents could select more than one work setting. Long-term care included nursing home, assisted living facility, other long-term care facility, home health agency, or home health care settings. The other clinical setting category included dentist office or dental clinic, pharmacy, emergency medical services, and other settings where clinical care or related services were provided to patients. ^§^ Respondents were recruited from two preexisting national opt-in internet sources: Medscape, a medical website managed by WebMD Health Professional Network, and Dynata, for general population internet panels.

During the 2024–25 respiratory virus season, 76.3% of HCP received an influenza vaccination, similar to the 2023–24 season (Table). Among all HCP, influenza vaccination coverage remained stable from the 2015–16 season through the 2019–20 season (average = 79.5%; range = 78.4%–80.6%) (Figure 1). Coverage declined to 75.7% during the 2020–21 season, increased slightly to 80.6% in 2021–22, declined the following season, and remained stable in subsequent seasons, although these differences were not statistically significant. Influenza vaccination coverage during the 2024–25 season was highest among HCP working in hospital settings (88.3%) and lowest among HCP working in LTC settings (70.5%) (Table). Coverage among assistants and aides (69.0%), other clinical personnel (76.1%), nonclinical personnel (76.5%), and nurses (79.8%) was lower than that among physicians (92.6%).

**TABLE:** Influenza and COVID-19 vaccination* coverage among health care personnel, by selected demographic and workplace characteristics — internet panel surveys,^†^ United States, 2023–24 and 2024–25 respiratory virus seasons

Characteristics and vaccine received	2023–24 season		2024–25 season		Percentage point change in weighted % vaccinated, 2023–24 to 2024–25 (95% CI)
	No. (weighted %)	Weighted % vaccinated (95% CI)^§^	No. (weighted %)	Weighted % vaccinated (95% CI)^§^	
**Influenza vaccine, total**	**2,750**	**75.4 (72.4 to 78.3)**	**2,648**	**76.3 (73.1 to 79.3)**	**0.9 (−3.3 to 5.1)**
**Age group, yrs**					
18–29 (Ref)	167 (12.7)	72.5 (61.5 to 81.8)	125 (13.7)	73.2 (61.1 to 83.2)	0.7 **(**−13.6 to 15.1)
30–44	1,071 (45.5)	74.8 (69.9 to 79.3)	1,018 (44.1)	75.2 (69.9 to 80.0)	0.4 (−6.4 to 7.1)
45–59	985 (27.9)	74.9 (70.0 to 79.5)	998 (28.5)	78.1 (73.1 to 82.6)	3.2 (−3.3 to 9.8)
≥60	525 (13.8)	80.7 (72.4 to 87.4)	507 (13.7)	79.3 (72.0 to 85.4)	−1.4 (−11.0 to 8.3)
**Race and ethnicity^¶^**					
White, non-Hispanic (Ref)	1,701 (58.9)	75.9 (72.6 to 79.0)	1,641 (56.3)	74.2 (70.2 to 77.9)	−1.7 (−6.7 to 3.2)
Asian, non-Hispanic	220 (6.7)	89.2 (80.7 to 94.8)**	210 (7.0)	89.2 (72.7 to 97.5)**	0 (−12.8 to 12.9)
Black or African American, non-Hispanic	322 (16.9)	70.5 (59.9 to 79.7)	338 (17.5)	76.5 (68.8 to 83.2)	6.0 (−5.7 to 17.8)
Hispanic or Latino	420 (14.5)	74.5 (64.4 to 82.9)	344 (14.9)	75.0 (62.6 to 84.9)	0.5 (−13.3 to 14.3)
Other, non-Hispanic	81 (3.0)	66.4 (49.2 to 80.9)^††^	114 (4.3)	85.2 (72.9 to 93.4)**	18.9 (1.0 to 36.7)^§§^
**Sex**					
Female (Ref)	1,911 (77.1)	72.5 (68.9 to 75.9)	1,827 (76.5)	76.0 (72.3 to 79.4)	3.5 (−1.4 to 8.3)
Male	817 (22.9)	84.8 (79.0 to 89.5)**	821 (23.5)	77.4 (70.2 to 83.6)	−7.4 (−15.6 to 0.8)
**Education**					
Some college education or less (Ref)	635 (28.9)	59.5 (53.5 to 65.3)	520 (26.2)	66.6 (60.0 to 72.7)	7.1 (−1.3 to 15.6)
Associate or bachelor’s degree	858 (43.9)	80.8 (75.6 to 85.3)**	782 (44.5)	78.5 (73.1 to 83.4)**	−2.2 (−9.1 to 4.6)
Master’s, professional, or doctoral degree	1,254 (27.2)	83.7 (79.1 to 87.5)**	1,346 (29.4)	81.7 (77.0 to 85.7)**	−2.0 (−7.8 to 3.8)
**Occupation^¶¶^**					
Physician (Ref)	355 (3.8)	93.0 (88.5 to 96.2)	366 (3.6)	92.6 (88.0 to 95.8)	−0.4 (−5.6 to 4.7)
Nurse practitioner/Physician assistant***	219 (1.7)	85.7 (79.9 to 90.3)**	244 (2.1)	88.0 (83.0 to 91.9)	2.3 (−4.3 to 8.8)
Nurse	220 (17.8)	87.6 (80.7 to 92.7)	195 (17.3)	79.8 (69.2 to 88.0)**	−7.8 (−18.3 to 2.8)
Pharmacist	328 (1.4)	93.9 (90.5 to 96.3)	315 (1.3)	94.6 (91.5 to 96.8)	0.8 (−2.9 to 4.4)
Other clinical personnel^†††^	592 (21.4)	81.8 (76.9 to 86.0)**	579 (21.5)	76.1 (70.5 to 81.2)**	−5.6 (−12.4 to 1.1)
Assistant/Aide	698 (23.5)	63.2 (58.5 to 67.7)**	621 (23.6)	69.0 (63.5 to 74.1)**	5.8 (−1.1 to 12.6)
Nonclinical personnel^§§§^	307 (30.3)	69.5 (61.5 to 76.8)**	298 (30.5)	76.5 (69.0 to 83.0)**	7.0 (−3.0 to 17.0)
**Work setting^¶¶¶^**					
Hospital	929 (38.8)	89.1 (84.8 to 92.5)**	1,030 (40.1)	88.3 (83.4 to 92.2)**	−0.8 (−6.4 to 4.8)
Ambulatory care	991 (37.4)	74.6 (69.8 to 79.0)	987 (36.3)	74.2 (68.7 to 79.2)	−0.4 (−7.2 to 6.4)
LTCF/Home health care****	660 (26.5)	65.2 (58.0 to 71.9)**	611 (27.9)	70.5 (64.4 to 76.1)**	5.3 (−3.5 to 14.1)
Other clinical setting^††††^	573 (9.7)	68.0 (59.2 to 76.0)	508 (11.6)	73.7 (63.6 to 82.3)	5.7 (−6.4 to 17.8)
**Location of primary workplace^§§§§^**					
Rural (Ref)	370 (13.8)	70.9 (61.3 to 79.2)	359 (13.1)	71.0 (62.3 to 78.8)	0.2 (−11.5 to 11.9)
Nonrural	2,376 (86.2)	76.1 (72.9 to 79.2)	2,289 (86.9)	77.1 (73.6 to 80.4)	1.0 (−3.6 to 5.5)
**U.S. Census Bureau region^¶¶¶¶^**					
Northeast (Ref)	620 (18.0)	73.2 (64.9 to 80.5)	559 (18.7)	79.6 (71.6 to 86.2)	6.4 (−3.8 to 16.6)
Midwest	588 (24.1)	80.4 (73.0 to 86.6)	583 (22.7)	76.5 (70.1 to 82.1)	−3.9 (−12.6 to 4.7)
South	985 (35.6)	74.7 (70.3 to 78.8)	1,002 (37.2)	76.7 (71.4 to 81.5)	2.0 (−4.4 to 8.4)
West	552 (22.3)	72.9 (65.9 to 79.2)	504 (21.4)	72.6 (64.1 to 80.1)	−0.2 (−10.3 to 9.8)
Employer vaccination requirement*****					
Required (Ref)	979 (39.3)	97.5 (95.9 to 98.7)	1,017 (38.7)	97.3 (95.4 to 98.6)	–0.2 (–2.2 to 1.7)
Recommended	1,089 (38.5)	74.4 (69.4 to 79.0)**	1,045 (39.8)	73.9 (68.0 to 79.3)**	−0.5 (–7.4 to 7.0)
Not required or recommended	650 (22.2)	37.8 (31.6 to 44.3)**	556 (21.4)	42.6 (35.2 to 50.2)**	4.8 (−4.7 to 14.4)
**Offered influenza vaccine on-site^†††††^**					
Yes (Ref)	967 (54.9)	74.2 (68.6 to 79.3)	977 (59.2)	73.0 (67.1 to 78.4)	−1.2 (−8.8 to 6.3)
No	773 (45.1)	45.0 (39.1 to 51.0)**	624 (40.8)	48.4 (41.5 to 55.4)**	3.4 (−5.5 to 12.3)
**Receipt of COVID-19 vaccine**					
Yes (Ref)	963 (31.3)	94.4 (91.8 to 96.3)	1,110 (40.2)	94.2 (91.6 to 96.2)	−0.1 (−3.2 to 2.9)
No	1,786 (68.7)	66.8 (62.8 to 70.6)**	1,536 (59.8)	64.3 (59.6 to 68.9)**	−2.5 (−8.4 to 3.5)
**COVID-19 vaccine, total**	**2,749**	**31.3 (28.3 to 34.5)**	**2,647**	**40.2 (36.8 to 43.7)**	**8.9 (4.3 to 13.4)^§§^**
**Age group, yrs**					
18–29 (Ref)	167 (12.7)	30.1 (19.6 to 42.3)	125 (13.7)	44.1 (31.9 to 56.9)	14.1 (−2.2 to 30.3)
30–44	1,071 (45.6)	28.5 (23.9 to 33.4)	1,016 (44.1)	38.2 (32.7 to 43.9)	9.7 (2.5 to 16.9)^§§^
45–59	985 (27.9)	29.8 (24.6 to 35.4)	999 (28.5)	40.3 (34.5 to 46.2)	10.5 (2.7 to 18.2)^§§^
≥60	524 (13.8)	45.0 (37.0 to 53.2)**	507 (13.7)	42.6 (35.0 to 50.6)	−2.3 (−13.3 to 8.6)
**Race and ethnicity^¶^**					
White, non-Hispanic (Ref)	1,700 (58.9)	29.6 (25.8 to 33.6)	1,640 (56.3)	35.5 (31.5 to 39.7)	5.9 (0.4 to 11.5)^§§^
Asian, non-Hispanic	220 (6.7)	53.4 (39.3 to 67.0)**	210 (7.0)	49.6 (35.5 to 63.8)	−3.8 (−22.8 to 15.3)
Black or African American, non-Hispanic	322 (17.0)	29.7 (21.9 to 38.4)	339 (17.5)	47.5 (38.5 to 56.6)**	17.8 (6.0 to 29.7)^§§^
Hispanic or Latino	420 (14.5)	28.7 (20.8 to 37.7)	344 (14.9)	45.8 (34.9 to 57.0)	17.1 (3.8 to 30.5)^§§^
Other, non-Hispanic	81 (3.0)	37.2 (19.9 to 57.2)^††^	113 (4.3)	40.6 (23.3 to 59.9)^††^	3.5 (−21.6 to 28.5)
**Sex**					
Female (Ref)	1,911 (77.1)	28.3 (25.0 to 31.8)	1,827 (76.5)	40.1 (36.3 to 44.0)	11.8 (6.7 to 16.9)^§§^
Male	816 (22.9)	40.1 (32.7 to 47.9)**	820 (23.5)	40.6 (33.4 to 48.0)	0.4 (−9.7 to 10.6)
**Education**					
Some college education or less (Ref)	635 (28.9)	25.4 (20.1 to 31.2)	521 (26.2)	36.2 (30.0 to 42.7)	10.8 (2.6 to 19.0)^§§^
Associate or bachelor’s degree	858 (43.9)	30.4 (25.2 to 35.9)	782 (44.5)	37.9 (32.1 to 44.0)	7.6 (−0.2 to 15.3)
Master’s, professional, or doctoral degree	1,253 (27.2)	39.1 (33.7 to 44.7)**	1,344 (29.3)	47.3 (41.5 to 53.1)**	8.2 (0.4 to 16.0)^§§^
**Occupation^¶¶^**					
Physician (Ref)	354 (3.8)	52.7 (45.5 to 59.8)	366 (3.6)	46.7 (40.0 to 53.5)	−6.0 (−15.6 to 3.5)
Nurse practitioner/Physician assistant***	219 (1.7)	29.4 (23.1 to 36.3)**	244 (2.1)	32.4 (26.0 to 39.3)**	3.0 (−6.1 to 12.1)
Nurse	220 (17.8)	29.8 (21.6 to 39.0)**	195 (17.3)	26.8 (18.6 to 36.4)**	−2.9 (−14.9 to 9.1)
Pharmacist	328 (1.4)	39.8 (33.5 to 46.5)**	314 (1.3)	41.3 (34.4 to 48.5)	1.5 (−7.8 to 10.8)
Other clinical personnel^†††^	592 (21.4)	34.1 (28.0 to 40.6)**	578 (21.5)	34.7 (28.4 to 41.4)**	0.6 (−8.1 to 9.4)
Assistant/Aide	698 (23.5)	27.7 (23.5 to 32.3)**	622 (23.6)	46.7 (41.1 to 52.3)	19.0 (12.1 to 25.9)^§§^
Nonclinical personnel^§§§^	307 (30.3)	30.1 (23.2 to 37.7)**	298 (30.6)	46.1 (38.3 to 54.0)	16.0 (5.6 to 26.3)^§§^
**Work setting^¶¶¶^**					
Hospital	929 (38.8)	33.9 (28.6 to 39.6)	1,029 (40.1)	44.1 (38.2 to 50.2)	10.2 (2.2 to 18.1)^§§^
Ambulatory care	990 (37.4)	26.1 (21.7 to 30.8)**	985 (36.3)	39.1 (33.4 to 44.9)	13.0 (5.8 to 20.1)^§§^
LTCF/Home health care****	660 (26.5)	34.8 (28.4 to 41.6)	612 (27.9)	44.5 (38.7 to 50.4)	9.7 (1.1 to 18.3)^§§^
Other clinical setting^††††^	573 (9.7)	28.1 (20.1 to 37.3)	507 (11.6)	40.6 (30.3 to 51.5)	12.4 (−0.7 to 25.6)
**Location of primary workplace^§§§§^**					
Rural (Ref)	370 (13.8)	25.7 (17.2 to 35.8)	359 (13.1)	30.3 (21.7 to 40.1)	4.6 (−7.9 to 17.2)
Nonrural	2,375 (86.2)	32.2 (28.9 to 35.6)	2,288 (86.9)	41.7 (37.9 to 45.6)**	9.5 (4.5 to 14.5)^§§^
**U.S. Census Bureau region^¶¶¶¶^**					
Northeast (Ref)	620 (18.0)	32.8 (26.1 to 40.1)	560 (18.8)	37.5 (30.1 to 45.3)	4.7 (−5.3 to 14.7)
Midwest	588 (24.1)	33.3 (26.0 to 41.1)	582 (22.7)	37.1 (30.5 to 44.2)	3.9 (−6.0 to 13.7)
South	984 (35.6)	27.7 (23.1 to 32.7)	1,001 (37.2)	42.0 (36.2 to 47.9)	14.3 (6.9 to 21.6)^§§^
West	552 (22.3)	33.7 (26.9 to 41.0)	504 (21.4)	42.7 (34.3 to 51.5)	9.0 (−1.8 to 19.8)
Employer vaccination requirement*****					
Required (Ref)	265 (12.5)	55.6 (45.0 to 65.7)	277 (14.1)	82.8 (73.3 to 90.0)	27.3 (14.5 to 40.0)^§§^
Recommended	1,293 (47.9)	38.0 (33.0 to 43.3)**	1,205 (44.7)	46.0 (40.6 to 51.6)**	8.0 (0.6 to 15.4)^§§^
Not required or recommended	1,160 (39.6)	15.5 (12.3 to 19.0)**	1,135 (41.3)	19.1 (15.2 to 23.6)**	3.7 (−1.5 to 8.8)
**Offered COVID-19 vaccine on-site^†††††^**					
Yes (Ref)	712 (26.1)	40.8 (33.8 to 48.2)	1,106 (46.2)	42.9 (37.0 to 49.0)	2.1 (−7.0 to 11.2)
No	1,741 (73.9)	23.2 (19.9 to 26.8)**	1,234 (53.8)	24.7 (20.7 to 29.1)**	1.5 (−3.8 to 6.8)
**Receipt of influenza vaccine**					
Yes (Ref)	2,158 (75.4)	39.2 (35.4 to 43.1)	2,135 (76.4)	49.6 (45.6 to 53.7)	10.4 (4.9 to 15.9)^§§^
No	591 (24.6)	7.2 (4.7 to 10.5)**	511 (23.6)	9.8 (6.4 to 14.2)**	2.6 (−2.0 to 7.2)

**Abbreviations**: LTCF = long-term care facility; Ref = referent group.

* Respondents were considered to have received a 2024–25 COVID-19 vaccine if they responded “Yes” to the question, “The 2024–25 COVID-19 vaccine became available on August 22, 2024. Have you received a COVID-19 vaccine since August 22, 2024?” Two respondents for influenza vaccination analysis and three respondents for COVID-19 vaccination analysis were removed due to unknown vaccination status.

^†^ Respondents were recruited from two preexisting national opt-in internet sources: Medscape, a medical website managed by WebMD Health Professional Network, and Dynata for general population internet panels.

^§^ Modified Clopper-Pearson 95% CI according to the approach of Korn and Graubard.

^¶^ Race and ethnicity were self-reported. Respondents who identified as Hispanic or Latino might be of any race. The “other” race category included persons who identified as American Indian or Alaska Native, Native Hawaiian or Pacific Islander, or Middle Eastern or North African, and persons who selected multiple races.

** Statistically significant (p<0.05) difference compared with Ref.

^††^ Estimates do not meet the CDC standards of reliability and should be interpreted with caution.

^§§^ Statistically significant (p<0.05) difference across seasons.

^¶¶^ Excluded students.

*** For the 2025 survey, nurse midwife, and nurse anesthetist were separated from the nurse occupation and regrouped with nurse practitioner and physician assistant for weighting purposes.

^†††^ Includes dentists, allied health professionals, technicians and technologists, emergency technicians, emergency medical technicians, and paramedics.

^§§§^ Includes administrative support staff members, administrative managers, and nonclinical support staff members.

^¶¶¶^ Respondents could select more than one work setting. Each work setting is represented by a separate variable with two values (yes and no; no is the Ref).

**** Nursing home, assisted living facility, other LTCF, home health agency, or home health care.

^††††^ Includes dentist office or dental clinic, pharmacy, emergency medical services, and other settings where clinical care or related services were provided to patients.

^§§§§^ Rurality was defined using zip code areas in which >50% of the population lives in a nonmetropolitan county, a rural U.S. Census Bureau tract, or both, according to the Health Resources and Services Administration’s definition of rural population.

^¶¶¶¶^ U.S. Census Bureau Regions and Divisions of the United States

***** Students were not asked workplace vaccination policy questions and were excluded.

^†††††^ Excluded students and those with employer vaccination requirement.

**FIGURE 2:**
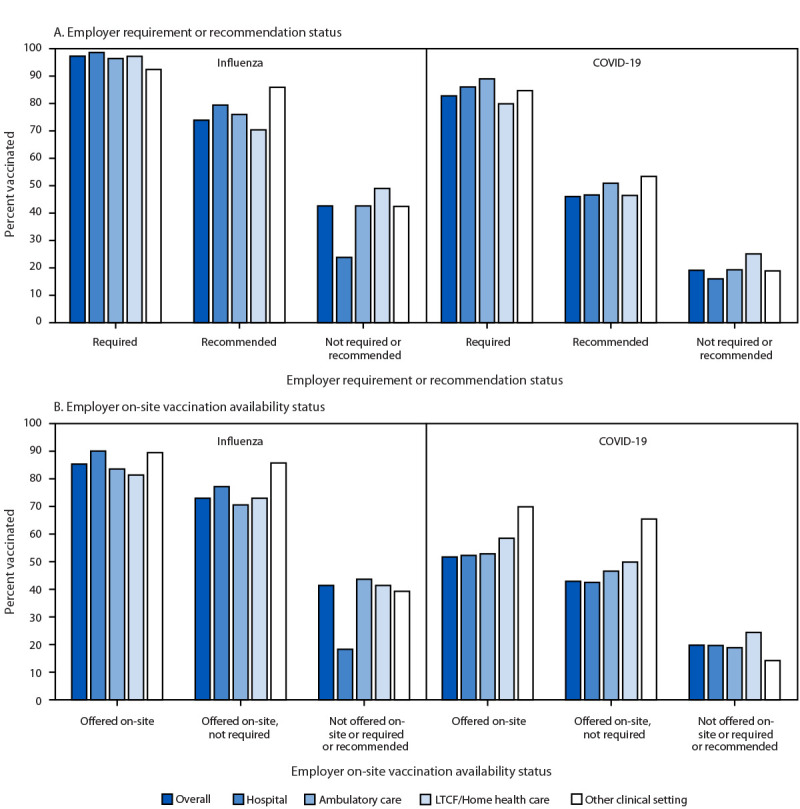
Percentage of health care personnel who received influenza and COVID-19 vaccinations,* by employer requirement or recommendation for vaccination (A) and offer for on-site vaccination^†^ (B), by work setting^§^ — internet panel surveys,^¶^ United States, 2024–25 respiratory virus season **Abbreviation**: LTCF = long-term care facility. * Respondents were asked, “Since July 1, 2024, has your employer recommended or required that you be vaccinated for flu?” and “Has your employer recommended or required that you be vaccinated with the 2024–25 COVID-19 vaccine?” Respondents who reported working in more than one location were asked separately for each work location. ^†^ Respondents were asked, “Since July 1, 2024, has your employer offered flu vaccinations on-site?” and “Does your employer offer the 2024–25 COVID-19 vaccine on-site?“ Respondents who reported working in more than one location were asked separately for each work location. ^§^ Respondents could select more than one work setting. Long-term care and home health care includes nursing home, assisted living facility, other LTCF, home health agency, or home health care. The other clinical setting includes dentist office or dental clinic, pharmacy, emergency medical services, and other settings where clinical care or related services were provided to patients. ^¶^ Respondents were recruited from two preexisting national opt-in internet sources: Medscape, a medical website managed by WebMD Health Professional Network, and Dynata for general population internet panels.

During the 2024–25 season, significantly higher influenza vaccination coverage was reported among non-Hispanic Asian HCP (89.2%) and non-Hispanic other HCP (85.2%); among those with a master’s, professional, or doctoral degree (81.7%); and those with an associate or bachelor’s degree (78.5%) compared with their respective reference groups. Coverage was higher among HCP who reported an employer requirement for influenza vaccination (97.3%) than among those who reported an employer recommendation (73.9%) or no employer recommendation or requirement for vaccination (42.6%) (Figure 2).

### COVID-19 Vaccination Coverage

Overall, 40.2% of HCP reported receiving the 2024–25 COVID-19 vaccine, a significantly higher percentage than the 31.3% who reported receiving it during the 2023–24 season (Table). Increases in COVID-19 vaccination coverage during the 2024–25 season ranged from 9.7 to 13.0 percentage points by work setting, compared with coverage during the 2023–24 season. Receipt of the COVID-19 vaccine was higher among non-Hispanic Black or African American (47.5%) HCP; those with a master’s, professional, or doctoral degree (47.3%); physicians (46.7%), assistants, and aides (46.7%); HCP who worked in nonrural settings (41.7%); and HCP in work settings where employers required COVID-19 vaccination (82.8%) and offered on-site vaccination (42.9%), compared with their respective reference groups.

### Employer Requirement for Vaccinations

Employer requirements for receipt of influenza and COVID-19 vaccination were reported by 38.7% and 14.1% of HCP, respectively (Supplementary Figure) (Figure 2). HCP working in other clinical settings and LTC settings were less likely to report requirements for influenza vaccination (17.1% and 24.7%, respectively) than were HCP working in ambulatory care settings (35.8%) and hospitals (59.1%). However, HCP working in ambulatory care settings were less likely to report requirements for COVID-19 vaccination (9.7%) than were those working in hospitals (15.5%) and LTC settings (19.3%).

In all work settings, coverage with both vaccines, by work setting, was higher among HCP who reported an employer requirement for vaccination (influenza: range = 92.4%–98.6% and 2024–25 COVID-19: range = 79.9%–89.0%) than among those whose employer neither recommended nor required vaccinations (influenza: range = 23.8%–49.0% and COVID-19: range = 16.0%–25.1%) (Figure 2). Coverage rates with influenza and 2024–25 COVID-19 vaccines were 73.0% and 42.9%, respectively, among HCP whose employer offered the vaccines on-site but did not require HCP vaccination; in contrast, among those HCP whose employers did not require, recommend, or offer these vaccines on-site, influenza and COVID-19 vaccination coverage rates were 41.4% and 19.8%, respectively.

## Discussion

During the 2024–25 respiratory virus season, influenza vaccination coverage among HCP was similar to that during the 2023–24 season; however, compared with coverage before the COVID-19 pandemic, influenza vaccination coverage has remained persistently low for the past three seasons. Influenza has been reported as a substantial cause of HCP absenteeism and disruption of health care services (*9*) and carries a risk for transmission to patients, coworkers, and others in the health care setting (*1*,*4*). Continued monitoring of influenza vaccination coverage among HCP is important for identifying causes for lower coverage since the 2018–19 season, and, among those HCP groups at higher risk for being unvaccinated, to help guide the development and implementation of evidence-based strategies to encourage vaccination, increase coverage, reduce influenza incidence among HCP and their patients, and limit strain on the health care system.

COVID-19 vaccination coverage among all HCP during the 2024–25 season increased significantly compared with coverage during the 2023–24 season. The 2024–2025 COVID-19 vaccine became available in August 2024, 1 month earlier than the 2023–24 COVID-19 vaccine became available the preceding year (*3*). Early availability of the vaccine might have given HCP more exposure to workplace vaccination policies and campaigns and permitted them more time to receive a COVID-19 vaccine. Aligning COVID-19 vaccine delivery, communication, and promotion with those for influenza vaccine has the potential to increase coverage with both vaccines during peak respiratory virus season (*10*).

Employer vaccination requirements, recommendations, and offers for on-site receipt of influenza and COVID-19 vaccination were strongly associated with influenza and COVID-19 vaccination coverage among HCP. Whereas requirements for COVID-19 vaccination were infrequently reported in all work settings, coverage with both influenza and COVID-19 was significantly higher when the vaccines were provided on-site, even in the absence of a vaccination requirement. These findings support the recommendations found in The Guide to Community Preventive Services, which include active promotion of on-site vaccination to increase influenza vaccination coverage among HCP.

Influenza vaccination coverage among HCP working in LTC settings is consistently lower than that among those working in other settings; however, during the 2024–25 season, influenza vaccination coverage among HCP working in LTC settings increased 5.3 percentage points compared with that during the 2023–24 season. Continuing to prioritize strategies and increasing activities to educate HCP about the benefits of vaccination might further increase influenza vaccination coverage among HCP in LTC settings (*1*,*2*,*4*).

In contrast to findings related to influenza vaccination coverage, COVID-19 vaccination coverage was highest among HCP working in LTC settings. The final rule published by the Centers for Medicare & Medicaid Services (CMS) requires CMS-certified nursing homes to offer COVID-19 vaccine to staff members as well as residents and to educate them about benefits and potential side effects, which might continue to increase vaccination coverage in these settings. A multipronged approach, including educating HCP about vaccination recommendations, coupled with employer vaccination requirements, recommendations, or on-site offer for vaccinations, might increase influenza and COVID-19 vaccination coverage.

### Limitations

The findings in this report are subject to at least five limitations. First, the study used a nonprobability sample of volunteer members of Medscape and Dynata internet panels. Responses were weighted to be representative of the U.S. population of HCP; however, results might not be generalizable to other HCPs. Second, the self-selection of respondents to the panels and to the survey might have introduced selection bias if participation in the panel or survey is associated with vaccination status. Third, vaccination status was self-reported and might be subject to recall or social desirability bias. Fourth, formal statistics that rely on the assumption of random sampling were used to identify differences in vaccination coverage among groups in this nonrandom sample, and results should be interpreted with caution; statistical organizations recommend transparency in methods when making inferences from nonprobability surveys. Finally, limited sample size resulted in coverage estimates in some subgroups that did not meet CDC reliability criteria for reporting proportions.

### Implications for Public Health Practice

Influenza and COVID-19 continue to cause considerable morbidity and mortality, and vaccination coverage among HCP remains low, increasing HCPs’ risk for illness, as well as the risk for persons under their care. Increasing vaccination coverage among HCP by offering on-site vaccination, especially ensuring that opportunities for influenza and COVID-19 vaccinations are aligned, and improving vaccine knowledge through provider education might lead to increased vaccination coverage, decreases in disease incidence among HCP, and subsequent decreased transmission to patients, particularly during the respiratory virus season and in LTC settings (Preventing Transmission of Viral Respiratory Pathogens in Healthcare Settings | CDC).

## Footnotes

* Physicians, nurse practitioners, physician assistants, nurses, dentists, pharmacists, allied health professionals, technicians, technologists, emergency medical technicians, paramedics, and students in a medical-related field were recruited from the current membership roster of Medscape.

† Assistants, aides, and nonclinical personnel (e.g., administrators, clerical support workers, janitors, food service workers, and housekeepers) were recruited from general population internet panels operated by Dynata.

§ Population control totals of U.S. HCP by occupation and work setting were obtained from the U.S. Department of Commerce Bureau of Labor Statistics’ occupational employment and wage statistics. Population control totals by other demographic characteristics were obtained from the Bureau of Labor Statistics’ labor force statistics from the current population survey.

¶ Major occupational categories included physicians and dentists, nurse practitioners and physician assistants, nurses, pharmacists, other clinical personnel (including allied health professionals, technicians and technologists, and emergency medical technicians and paramedics), assistants and aides, and nonclinical personnel (including administrative support staff members and managers and nonclinical support staff members).

** Respondents were considered to have received a 2024–25 COVID-19 vaccine if they responded “Yes” to the question, “The 2024–25 COVID-19 vaccine became available on August 22, 2024. Have you received a COVID-19 vaccine since August 22, 2024?”

†† Employer-related vaccination questions included, “Since July 1, 2024, has your employer recommended or required that you be vaccinated for flu?” and “Has your employer recommended or required that you be vaccinated with the 2024–25 COVID-19 vaccine?” Responses for both questions included “recommend,” “require,” “neither,” or “unsure.” Respondents could only select one option for each setting where they worked. Questions for on-site vaccination questions included, “Since July 1, 2024, has your employer offered flu vaccination on-site?” and “Does your employer offer the 2024–25 COVID-19 vaccine on-site?”

§§ 45 C.F.R. part 46; 21 C.F.R. part 56; 42 U.S.C. Sect. 241(d); 5 U.S.C. Sect. 552a; 44 U.S.C. Sect. 3501 et seq.

¶¶ A survey response rate requires specification of the denominator at each stage of sampling. During recruitment of an online opt-in survey sample, such as the internet panels described in this report, these data are not available; therefore, a response rate cannot be calculated. Instead, the survey completion rate is provided.
